# Suicidal ideation, psychopathology and associated factors among HIV-infected adults in Indonesia

**DOI:** 10.1186/s12888-020-02666-1

**Published:** 2020-05-24

**Authors:** Youdiil Ophinni, Adrian, Kristiana Siste, Martina Wiwie, Gina Anindyajati, Enjeline Hanafi, Reza Damayanti, Yoshitake Hayashi

**Affiliations:** 1 Department of Pathology, Graduate School of Medicine, Kobe University, Kobe, Japan; 2 Department of Psychiatry, Faculty of Medicine, Universitas Indonesia, 71 Diponegoro, Central Jakarta, DKI, Jakarta, Indonesia

**Keywords:** Depression, HIV/AIDS, Psychopathology, Suicidal behavior, Efavirenz

## Abstract

**Background:**

Suicidal behavior is a prevalent psychiatric emergency in HIV-infected adults. Detection of suicidal ideation is important in planning early psychiatric intervention and optimizing HIV/AIDS management. Characterization of suicidal ideation among HIV-infected adults is crucial; however, practically there is no data in Indonesia, the country with the second largest burden of HIV/AIDS epidemic in Asia. This study aims to identify suicidal ideation and analyze the associated psychopathology and determining factors among HIV-infected adults in Indonesia.

**Methods:**

An observational cross-sectional study was conducted among HIV-infected adults aged 18–65 years old receiving antiretroviral therapy (ART). Measurement using Symptom Checklist-90 (SCL-90) was performed to assess the existing psychopathology. Firth’s penalized logistic regression analysis was performed to identify factors associated with suicidal ideation.

**Results:**

A total of 86 subjects were recruited. Most subjects were male (65.1%), median age was 35 years, and median latest CD4 count was 463 cells/μl. Lifetime suicidal ideation was identified in 20 subjects (23.3%). Mean SCL-90 T-score for depressive and anxiety symptoms were both significantly higher among subjects with suicidal ideation (*M =* 60.75, *SD* = 12.0, *p* = 0.000 and *M* = 57.9, *SD* = 2.8, *p* = 0.001, respectively) compared to those without. Bivariate analyses showed that lifetime suicidal ideation was associated with depressive and anxiety symptoms, non-marital status, CD4 count < 500 cells/μl, and efavirenz use. Multivariate analysis identified that a single-point increase in SCL-90 depression symptoms score (AOR 1.16, 95% CI 4.5–123.6, *p* = 0.000) and efavirenz use (AOR 5.00, 95% CI 1.02–24.6, *p =* 0.048) were significant independent factors related to suicidal ideation.

**Conclusion:**

Suicidal ideation is commonly found among Indonesian HIV-infected adults on ART. Depressive symptoms and efavirenz use are independent factors related to the presence of suicidal ideation. Thus, early screening of psychopathology as well as substitution of efavirenz with other ART regiment are recommended to prevent suicide and improve HIV/AIDS management outcome.

## Background

Suicide is one of the leading causes of death worldwide. In 2018, World Health Organization (WHO) estimated that almost 800,000 people die due to suicide annually, 79% of which occurred in low- and middle-income countries (LMICs) [1, 2]. Phases of suicidal behavior can be divided into suicidal ideation, plans, and attempt [3, 4]. Centers for Disease Control and Prevention (CDC) defines suicide as death caused by self-directed harmful behavior with an intention of dying as a result, while suicidal ideation is defined as having a thought of or considering suicide [3, 5, 6]. WHO surveyed 21 countries in 2001–2007 and reported lifetime prevalence of suicidal ideation as 2.0 to 2.1% [7, 8]. The majority of individuals (60%) who made a suicidal plan or attempt proceeded to do so within one year since the ideation onset [3, 7].

Suicidal behavior has been well characterized as a pervasive comorbidity among people living with HIV (PLHIV), which may represent the prevalence of mental disorders among PLHIV [9]. HIV infection may either directly disrupt central nervous system (CNS), associated with CNS-related opportunistic infections that defines acquired immune deficiency syndrome (AIDS), or might be accompanied by environmental issues negatively affecting mental health (e.g. substance use, socioeconomic burden, and stigma) [10–12]. All of these may lead to increased risk of psychopathology. Indeed, a 2018 study in UK showed that a higher proportion of PLHIV presented with depressive symptoms (27.0% versus 10.5%) and anxiety (21.6% versus 9.6%) compared to demographically-matched HIV-negative group [13, 14]. Almost double of those numbers were reported in Asian countries, such as China (50.8% of PLHIV showing depressive symptoms) and India (50% and 25–36% of PLHIV showing depressive and anxiety symptoms, respectively) [15–17]. Prevalence in LMICs was similarly high, but resource limitation further added difficulties to the situation, due to underdiagnosis of mental disorders, suboptimal access to treatment, and limited research-based knowledge [18, 19]. Subsequently, suicidal behavior was reported at an alarmingly high rate among PLHIV: 22.5% of PLHIV in Ethiopia showed suicidal ideation [20], increase in suicide rate due to HIV seropositive status was reported in South Africa (13.3 to 18.9%) [21], and suicidal ideation was reported in as many as 45% of HIV-positive men who have sex with men (MSM) in India [22]. High suicidal burden among PLHIV has been consistently reported across countries, highlighting the urgency to monitor associated factors to suicidal ideation [23].

Notwithstanding the scarcity of studies in LMICs, distinct factors predictive of suicidal behavior have been well described [7, 18, 19]. HIV positive status, together with socioeconomic burden, poor social support, and perceived stigma, were found to be predictive of suicide attempt in South Africa [21]. In high-income countries (HICs), socioeconomic factors are associated less strongly compared to the progression of HIV disease itself. In Switzerland, for example, lower cluster-of-differentiation-4-expressing T cells (CD4 T cells) and advanced HIV/AIDS stage were related to higher suicide rate among PLHIV [24]. In particular, antiretroviral therapy (ART) containing efavirenz as a non-nucleoside reverse transcriptase inhibitor (NNRTI), was linked to significant adverse effects on the CNS which may induce psychiatric symptoms, such as severe depression and suicidal ideation [25]. PLHIV receiving efavirenz were approximately twice as likely to experience suicidal thoughts or attempt suicide compared to those receiving other medications [26]. On the other hand, non-HIV-related risk factors did not vary between countries, which included female gender, young adult (18–34 years old), low educational level (secondary education or lower), married, and having a mental disorder [7]. While diagnosable mental disorders prior to suicidal behavior are more prevalent in HICs (> 90% versus 60%), psychopathologies are as predictive of suicidal ideation in LMICs as they are in HICs [2, 27, 28]. Ultimately, psychopathology in PLHIV was found to affect the clinical outcome and mortality, adherence to ART, HIV risk behaviors, and quality of life (QOL) poorly [11, 17, 29, 30]. Psychological interventions (e.g. cognitive behavioral therapy and group psychotherapy) in HICs are effective in improving depressive symptoms among PLHIV, as evidently shown by clinical trials [31–33]. Such studies also highlighted the importance of early detection of suicidal behavior in order to enable timely intervention to improve mental health and HIV/AIDS-related treatment outcomes [34]. Nevertheless, more data are urgently needed in LMICs, including Indonesia.

Indonesia is the third most populous LMICs in the world and known to have the second largest burden of PLHIV in Asia [35, 36]. Although the suicide rate among general population in Indonesia (3.4 per 100,000 people) is lower than the crude suicide rate in both South-East Asia and worldwide (13.2 and 10.6 per 100,000 people, respectively), suicide is regarded as a major concern in Indonesia [1]. Suicidal ideation was reported in 4.75% of Indonesian adolescents [37], while a 2019 survey of 1018 adults in the general population reported that 1 of 4 subjects experienced suicidal thoughts [38]. With regard to PLHIV in Indonesia, however, the characteristic of suicidal ideation is practically unknown. In this study, we aim to identify suicidal ideation and psychopathology among PLHIV in Indonesia and the associated factors. Prior evidences in other countries signified depression as a major factor to the emergence of suicidal ideation among PLHIV [20, 39–41]. Thus, we hypothesize that Indonesian PLHIV who have experienced suicidal ideation will show significantly higher depression symptoms score. It is hoped that the result of this study is beneficial to optimize early psychological intervention, prevent suicide, and improve the overall HIV/AIDS treatment outcome in Indonesia.

## Methods

### Demographics and data collection

A cross-sectional study was conducted among subjects who met inclusion criteria, which were adult PLHIV aged 18–65 years old receiving antiretroviral therapy (ART) and fluent in Indonesian language. Subjects were excluded if severe mental disorders, including psychotic disorders as well as intellectual and developmental disabilities, were revealed during psychiatric examination. Power analysis with medium effect size (*d* = 0.50) and an alpha of 0.05 showed that a minimum size of 86 subjects was necessary to achieve a power of 0.90. Subject recruitment was done by consecutive sampling and performed from July to October 2017 in HIV Integrated Service Unit of dr. Cipto Mangunkusumo General Hospital, Jakarta—the largest outpatient clinic for HIV management and research center in Indonesia, which also serves as the national referral center for HIV/AIDS cases all over the country.

Subjects were requested to fill out measurement questionnaire and sociodemographic survey. Whenever the subjects could not independently fill in the questionnaires, interviews were done instead by three interviewers under the supervision of a licensed psychiatrist. Assessment of suicidal ideation was done via direct interview, while HIV-related data (symptoms of fever, fatigue, pharyngitis, lymphadenopathy, myalgia, and chronic diarrhea with the results of a positive enzyme-linked immunosorbent assay [ELISA] examination) were procured from medical records. Written informed consents were obtained from all recruited subjects, confidentiality of the subjects’ responses was ensured, and permission to retrieve data from subjects’ medical record was also obtained.

### Measurements

Sociodemographic data on gender, age, residence, education, monthly income (with brackets based on *Riset Kesehatan Dasar* or Indonesian National Health Survey in 2013), religion, ethnic group, marital status, and presence of cohabitant were collected. HIV-related data including duration of HIV diagnosis, latest CD4 count, ART regiment, ART duration, and presence of opportunistic infection were also obtained. CD4 count is the number of CD4-expressing T cells per microliter of blood, with normal range of 500–1400 cells/μL—CD4 cells has important roles in adaptive immune systems and are the main cells destroyed by HIV. ART regiments were defined as the last antiretroviral drugs taken to suppress HIV replication inside the body.

Finally, lifetime suicidal ideation was recorded if the subjects responded with “*yes*” to the following question, “*Have you ever seriously thought about committing suicide?*” Similar method of assessment was done in other studies [8, 20, 42]. The question was taken from a section of World Mental Health-Composite International Diagnostic Interview (WMH-CIDI) suicidality assessment [43].

### Symptoms check list 90 (SCL-90) questionnaire

The subscales of depression and anxiety of the Symptoms Check List 90 (SCL-90) questionnaire, translated into Indonesian, were used. SCL-90 is a self-report psychometric instrument that is used to quantitatively assess mental status, consisting of 90 items, each with five-point Likert scale: ‘not at all’, ‘a little bit’, ‘moderately’, ‘quite a bit’, and ‘extremely’. The instrument is divided into nine psychiatric symptom and psychological distress subscales. SCL-90 was translated into Indonesian and validated by Herianto et al. with good validity and reliability. Reliability test revealed a Cronbach’s alpha of 0.67 with the highest of 0.94 on the depression subscale. The Indonesian version of SCL-90 was conducted using cut-off T-score of > 60 to screen psychopathologies, yielding sensitivity of 82.92%, specificity of 83%, positive predictive value of 80.0%, and negative predictive value of 84.7% [44].

### Statistical analyses

Statistical analyses were conducted using STATA 14 (StataCorp, TX, USA). Chi-square or t-tests were done for bivariate analyses, and 2-tailed *p* value of less than 0.05 was considered significant. Age as well as depressive and anxiety symptoms as measured by SCL-90 were regarded as continuous variables, while the rest of covariates were nominal variables. Due to the low number of recruited subjects, multivariate analysis was conducted using Firth’s penalized logistic regression (*firthlogit* command in STATA) which shrinks the regression coefficient towards zero, for variables with *p* < 0.25 in the bivariate analyses.

## Results

### Baseline demographics

A total of 86 subjects met the inclusion criteria. All approached subjects agreed to participate in the study and completed the questionnaire without any missing data. Most recruited subjects were male (65.1%) with median age of 35 years (Table 1). Most subjects came from relatively low socioeconomic strata, in which the majority were high school graduates (44.2%) and had monthly income less than IDR 1,500,000 (53.5%, equivalent to USD ~ 107). More than half of subjects were not married (53.5%) and living together with their family (67.4%).

**Table 1:** Demographic characteristics of study subjects (*N* = 86)

Variables	Frequency (%)
**General demographics**	
**Gender**	
Male	56 (65.1)
Female	30 (34.9)
**Age (years)**	
Median, IQR	35.0, 31.3–38.0
18–34	40 (46.5)
35–65	46 (53.5)
**Residence**	
Jakarta	56 (65.1)
Outside Jakarta	30 (34.9)
**Education**	
Middle school or lower	11 (12.8)
High school	38 (44.2)
Higher education	37 (43.0)
**Monthly income (IDR)**	
< 1,500,000 (< 107 USD)	46 (53.5)
≥ 1,500,000 (≥107 USD)	40 (46.5)
**Religion**	
Islam	60 (69.8)
Protestant	20 (23.3)
Catholic	3 (3.5)
Hinduism	1 (1.2)
Buddhism	2 (2.3)
**Ethnic group**	
Javanese	35 (40.7)
Sundanese	14 (16.3)
Betawi	5 (5.8)
Batak	6 (7.0)
Chinese-Indonesian	11 (12.8)
Others	15 (17.4)
**Marital status**	
Married	40 (46.5)
Single	32 (37.2)
Divorced	14 (16.3)
**Living together with**	
Alone	12 (14.0)
Partner	16 (18.6)
Family	58 (67.4)
**HIV-related**	
**Duration of HIV diagnosis (years)**	
Median, IQR	5, 2–9
< 2	25 (29.1)
≥ 2	61 (70.9)
**Latest CD4 count (cells/μl)**	
Median, IQR	463, 258–654
< 500	35 (40.7)
≥ 500	51 (59.3)
**ART regiment**	
Efavirenz-based	42 (48.8)
Other NNRTI-based	32 (37.2)
PI-based	12 (14.0)
**ART duration (years)**	
Median, IQR	4, 1–8
< 2	31 (36.1)
≥ 2	55 (63.9)
**Opportunistic infections**	
Present	31 (36.0)
Absent	55 (64.0)

*IQR* interquartile range, *IDR* Indonesian Rupiah, *USD* United States Dollar, *CD4* cluster of differentiation 4, *ART* antiretroviral therapy, *NNRTI* non-nucleoside reverse transcriptase inhibitor, *PI* protease inhibitor

Subjects were mostly PLHIV who had long-term treatment in the hospital, with median duration since HIV diagnosis of 5 years. Most subjects (51.2%) were using efavirenz-based regiment and median duration of ART use among all subjects was 4 years. HIV infection was generally well-controlled under therapy as most subjects (59.3%) had CD4 count of more than 500 cells/μl.

### Suicidal ideation, psychopathology and associated factors

Using a cut-off T-score of 61, the SCL-90 measurements identified 15 (17.4%) and 13 (15.1%) subjects with depression and anxiety symptoms, respectively. Presence of both depression and anxiety symptoms were identified in 10 (11.6%) subjects, while 68 (79.1%) subjects had neither.

Out of 86 subjects, lifetime suicidal ideation was identified in 20 (23.3%) (Table 2). Mean T-score for both depressive and anxiety symptoms were significantly higher among subjects with suicidal ideation (*M =* 60.75, *SD* = 12.0, *p* = 0.000 and *M* = 57.9, *SD* = 2.8, *p* = 0.001, respectively). Non-marital status was also an associated factor, since 12 out of 32 subjects who were single had suicidal ideation [*χ*^*2*^(1,*N* = 86) = 7.36, *p* = 0.009]. No association was found with the remaining demographical data, including age, gender, education, monthly income, and presence of cohabitant. Among HIV-related covariates, the latest CD4 count of less than 500 cells/μl was significantly associated with lifetime suicidal ideation [*χ*^*2*^(1,*N* = 86) = 8.67, *p* = 0.003]. Efavirenz use was also associated with suicidal ideation [*χ*^*2*^(1,*N* = 86) = 4.67, *p* = .031]. Duration since HIV diagnosis, duration of ART use, and presence of opportunistic infections were non-associative.

**Table 2:** Bivariate analyses between psychopathology and other determining factors with suicidal ideation

	With suicidal ideation (N, %)	Without suicidal ideation (N, %)	*p*	COR (95% CI)
**Psychopathology (T-score)**				
Depression (mean, SD)	60.8 (12.0)	46.7 (6.6)	**0.000**	1.16 (1.09–1.24)
Anxiety (mean, SD)	57.9 (2.8)	47.7 (1.0)	**0.001**	1.10 (1.04–1.17)
**Age** (median, IQR)	35.5 (32.5–38.5)	34 (30.0–37.0)	0.277	0.96 (0.89–1.03)
**Gender**				
Female	6 (30.0)	24 (36.4)	0.798	0.75 (0.26–2.21)
Male	14 (70.0)	42 (63.6)		
**Education**				
Middle school or lower	4 (20.0)	7 (10.6)	0.272	0.48 (0.12–1.83)
High school or higher	16 (80.0)	59 (89.4)		
**Monthly income**				
Low	14 (70.0)	32 (48.5)	0.152	2.48 (0.85–7.24)
Middle and high	6 (30.0)	34 (51.5)		
**Marital status**				
Single	12 (60.0)	20 (30.3)	**0.009**	5.40 (1.53–18.97)
Divorced	4 (20.0)	10 (15.2)	0.106	3.60 (0.76–17.01)
Married^a^	4 (20.0)	36 (54.5)		
**Living with**				
Nobody	4 (20.0)	8 (12.1)	0.289	
Family	16 (80.0)	58 (87.9)		1.81 (0.48–6.80)
**Latest CD4 count (cells/ul)**				
< 500	16 (80.0)	28 (42.4)	**0.003**	
≥ 500	4 (20.0)	38 (57.6)		5.43 (1.63–18.01)
**Opportunistic infections**				
Present	9 (45.0)	22 (33.3)	0.493	
Absent	11 (55.0)	44 (66.7)		1.64 (0.59–4.53)
**ART regiment**				
Efavirenz-based	14 (70.0)	28 (42.4)	**0.031**	
Not efavirenz-based	6 (30.0)	38 (57.6)		3.17 (1.08–9.27)
**Duration of HIV diagnosis**				
< 2 years	4 (20.0)	21 (31.8)	0.460	
≥ 2 years	16 (80.0)	45 (68.2)		0.54 (0.16–1.80)
**ART duration**				
< 2 years	5 (25.0)	26 (39.4)	0.364	
≥ 2 years	15 (75.0)	40 (60.6)		0.51 (0.17–1.58)
**Total**	20 (23.3)	66 (76.7)		

^a^reference group

Based on the results of bivariate analyses as well as prior evidences, the following covariates were included into penalized logistic regression model: age, gender, marital status, depression symptoms, anxiety symptoms, latest CD4 count, and efavirenz use (Table 3). Among all variables, depressive symptoms (*p* = 0.004, AOR 1.26, 95% CI 1.08–1.48) and efavirenz use (*p* = 0.048, AOR 5.00, 95% CI 1.02–24.6) were identified as the significant factors related to the presence of lifetime suicidal ideation.

**Table 3:** Multivariate analysis between selected determining factors with suicidal ideation

Covariate	*p*	AOR (95% CI)
Older age	0.052	0.89 (0.79–1.01)
Female gender	0.183	3.52 (0.55–22.47)
Non-marital status	0.069	4.28 (0.90–20.48)
1-point increase in SCL-90 depressive symptoms T-score	**0.004**	**1.26 (1.08–1.48)**
1-point increase in SCL-90 anxiety symptoms T-score	0.192	0.91 (0.79–1.05)
Latest CD4 < 500 cells/μl	0.057	3.35 (0.77–14.6)
Efavirenz use	**0.048**	**5.00 (1.02–24.6)**

## Discussion

We have identified the prevalence of lifetime suicidal ideation among Indonesian PLHIV to be 23.3%. This number is comparable to prior studies among PLHIV in both HICs and LMICs: 31% had current suicidal ideation among PLHIV in UK [45], 14% (suicidal ideation at any time point or lifetime) and 19% (at present time) in the United States [39, 46], 21% (in the past) in Australia [47], 14% (lifetime) in India [48], 22.5 to 33.6% (lifetime) in Ethiopia [20, 41], 8.8% (lifetime) in Uganda [49], and 28.8% (current) in South Africa [50].

We also analyzed independent covariates from demographic factors, psychopathology, and HIV-related variables; five were found associative to suicidal ideation—depressive symptoms, anxiety symptoms, single marital status, latest CD4 count of less than 500 cells/μl, and efavirenz use. Depression and anxiety symptoms are frequently discovered among PLHIV, and both manifestations have been linked strongly to the development of suicidal ideation [51, 52]. Depression among PLHIV can be precipitated by chronic HIV-related conditions, adverse effects of medication (e.g. efavirenz), as well as social pressures (e.g. difficulty of finding partner, jobs, and negative stigma from the community) [17, 53, 54]. Similar facets have been shown to trigger anxiety among PLHIV in LMICs [55–57]. Ultimately, depression and anxiety may lead to higher risk of suicidal ideation in PLHIV compared to those in HIV seronegative population [10, 40]. Prevalence of depression found in our study, however, was relatively low (17.4%), which may be caused by the psychometric weakness of SCL-90 or the small sample size. Nevertheless, depression prevalence rates among PLHIV were generally reported in many studies to be highly diverse (0–80%) due to differences in target subpopulation or instrument cut-off points [58, 59]. In this study, cut-off T-score of SCL-90 was set to be > 60, but eventually we included T-score in the regression model as a continuous variable. Subsequent multivariate analysis confirmed 1-point increase in depressive symptoms subscale as an independent factor, with AOR of 1.26, toward the presence of suicidal ideation.

We did not find association between age and suicidal ideation in PLHIV. This differs with findings in general population where the association between depression and suicidal behavior are stronger in the elderly population [60]. Furthermore, study among PLHIV by Schlebusch et al. in South Africa reported that HIV seropositive status, age, and gender were all associated with suicidal ideation [21]. The discrepancy is most likely due to the small sample size in our study. Gender also did not correlate with suicidal ideation in our study, in contrast to the report by Bitew et al. in Ethiopia in which female PLHIV were found to be 2.3 times more likely to have suicidal ideation compared to male PLHIV [41]. Gebremariam et al. discovered that female PLHIV in Ethiopia were more likely to have suicidal ideation due to daily burden as housewives and perceived negativity of depending on men [20]. Study in Brazil by Ceccon et al. also reported that 50% of female PLHIV had risk of suicidal ideation, which may relate to gender violence [61]. Non-associative result in our study was most likely due to limited sample size and subject variability. Survey by Ministry of Health of Indonesia in 2018 showed that the distribution of PLHIV in Indonesia was leaning towards male in a 2:1 ratio [62], and consecutive sampling method would naturally led to more male subjects. A targeted study towards female participants may further elucidate the association of gender with suicidal behavior; a study that is warranted in LMICs.

Several existing studies reported the correlation between low income and suicidal ideation in PLHIV. Onyebueke and Okwaraji described correlation between low income among PLHIV in Nigeria with depression and increased risk of suicidal ideation [63]. Financial constraints and difficulty in getting better jobs increases stigma, psychosocial stress, and risk of psychopathology [21, 63]. The only related data collected in this study was monthly income; further exploration into occupational status and economic QOL may further explain the role of socioeconomic status in suicidal behavior among PLHIV. Indeed, correlation of income with QOL has been robustly shown in both HICs and LMICs, affecting both mental health and peer perception, as well as stigma from the local community [64, 65].

Socio-environmental condition is heavily linked to mental health problems, including in PLHIV. Perception of solitude among PLHIV is significantly associated to depression and suicidal ideation as studied in several countries. For example, unmarried subjects had higher rate of depressive behavior in India [53], and solitary living condition among PLHIV in Ethiopia increased the likelihood of suicidal ideation by 13.5 times [41]. In our study, unmarried subjects had significantly higher rate of suicidal ideation in bivariate analyses while the presence of cohabitants was non-associative. Non-marital status and presence of cohabitants, however, may not correlate with the actual presence of social support. Exclusion and the feeling of being isolated from surroundings might directly relate to depression and suicidal thoughts—37% of PLHIV experienced loneliness and social isolation due to their HIV seropositive status, which correlated with depressive symptoms [66].

In our study, CD4 cell count of < 500 cells/μL was not independently associated with lifetime suicidal ideation. This is contrary with result by Bitew et al., who reported CD4 count < 500 cells/μL as a predictive factor of suicidal thoughts with AOR of 2.5 [41]. Amanor-Boadu et al. went further and defined CD4 count as a clinical marker of untreated depression in African-American PLHIV [67]. In progressive HIV disease, low CD4 count was associated with AIDS defining illness and increasing mortality, while untreated depression and poor ART adherence were independently associated with poorer CD4 outcomes [63]. Discrepancy in result might be due to the smaller sample size in our study compared to the aforementioned studies. Also in contrast to our study, Bitew et al. stated strong correlation between opportunistic infections, depression, and suicidal ideation [41]. Risk of suicide may increase due to severe physical conditions such as muscle wasting, or direct pathogenesis to the CNS via opportunistic infections or HIV-associated neurocognitive disorder (HAND), or vice versa—depression is also predictive of frailty phenotype and HAND [41, 68–70]. Opportunistic infections tend to be more severe as CD4 count decrease below 200 cells/μL; however, infections do not always occur in low CD4 state. Decline in immune status itself may negatively affect mental state and induce psychological frailty, rather than actual opportunistic infections to the CNS or other organ systems [12, 70]. In our study, most subjects already received ART for years which presumably led to relatively high median CD4 count and low occurrence of opportunistic infections. Recruitment of newly-diagnosed or untreated patients may further shed light on the correlation between suicidal behavior, immune dysfunction, and AIDS.

Regression model confirmed that the use of efavirenz-based ART regiment is associated to suicidal ideation in this study, with AOR of 5.00. Use of efavirenz has long been associated with CNS-related adverse effects including but not limited to dizziness, fatigue, nightmares, and insomnia [71, 72]. Specifically, long-term efavirenz use is associated with neuropsychiatric disorders, including depression and suicidal ideation lasting for several months [72, 73]. In 2015, Mothapo et al. reported that efavirenz discontinuation improved depressive symptoms as measured by SCL-90 [74]. Studies in animal models showed that efavirenz evidently disrupted brain mitochondrial P450 systems and neurochemical balance, particularly serotonin, both of which led to its neurotoxic effects [75, 76]. Yet, the link to depressive behavior remains controversial. Study by Mollan et al. concluded that efavirenz was associated with 2-fold increased risk of suicidality [26], while a newer study by Dabaghzadeh et al. in 2015 did not found any correlation [77] and Chang et al. in 2018 reported that suicidal thoughts did not occur more frequently in efavirenz recipients compared to another commonly used NNRTI, nevirapine [78]. The author opined that efavirenz is metabolically processed slower, resulting in lower peak levels and less CNS toxicity [78] while long duration of use is also known to induce tolerance towards CNS-related adverse effects [79]. Nevertheless, the specific association between efavirenz and suicidal ideation seems to be understudied. A lot of other studies with similar method to our study did not analyze the types of ART regiments used [20, 41]. Further studies are warranted.

The duration of both HIV diagnosis and ART use did not associate with suicidal ideation in this study. These data are supported by Dabaghzadeh et al. who evidently showed no correlation between duration of HIV diagnosis (median 24 months) and ART use (median 27 months) with suicidal ideation among PLHIV in Iran [77]. However, Olley et al. stated that patients who were diagnosed early with HIV might experience reduced stress over time after a period of adaptation [80]. Furthermore, study by Amiya et al. in Nepal added that stress associated with suicidal ideation arises early after HIV diagnosis due to inadaptation and absence of effective coping mechanism towards HIV seropositive status, which palliates over time [81]. Govender and Schlebusch also reported significant association between depression and feelings of hopelessness with HIV diagnosis of less than 6 weeks in South Africa [50]. Similar to low immune status, recruitment of newly-diagnosed PLHIV may better characterize the link between suicidal behavior to shorter duration of diagnosis and ART use.

### Strengths and limitations

This study is the first to identify suicidal ideation, associated psychopathology, and determining factors among PLHIV in Indonesia. We also included HIV-related covariates into the analyses, including the use of efavirenz as ART regiment, which was subsequently found to be a significant associated factor. This study also highlights the association between depressive symptoms and suicidal ideation among PLHIV, and thus, depression screening in HIV integrated care should be regarded as a mandatory service. However, since SCL-90 was a screening instrument, clinical diagnosis of psychiatric problems has to be reaffirmed by interview using diagnostic criteria. Reliance on SCL-90 as a diagnostic tool may introduce bias in the outcome of our study and inclusion of accurate psychiatric diagnosis in future studies may provide stronger link between HIV and psychopathologies.

A number of other limitations can be identified. The cross-sectional nature limits any establishment of causality between independent and dependent variables. Thorough observation on opportunistic infections and HIV clinical stages may better illustrate the link between physical and psychological comorbidities in PLHIV. Other potentially important socio-environmental variables were not assessed, e.g. social support, acceptance of HIV status, perceived stigma on HIV status, occupational status, and access to health care services. Lifetime instead of point prevalence was assessed, which only predicted the presence of suicidal ideation rather than its emergence. In addition, the ascertainment of suicidal ideation was done with a single question instead of a validated scale. Further study about characterization of suicidal attempt should be performed, which may enable an analysis of progression from ideation to realization. We also hope that further studies in LMIC could examine the implications specifically for women with HIV (e.g. [61]), and PLHIV with shorter duration of diagnosis and therapy. Most importantly, the small sample size of this study and lack of control group means that representation of results is not optimally accurate and therefore needs to be interpreted carefully. Longitudinal study with much larger sample size is warranted in Indonesia, as well as LMICs in general.

## Conclusion

We have identified the prevalence of suicidal ideation among Indonesian PLHIV on ART to be 23.3%. Suicidal ideation is significantly associated with the psychopathology of depressive symptoms, anxiety symptoms, single marital status, CD4 count < 500 cells/μl, and efavirenz use. Depressive symptoms and efavirenz use are independent factors associated with the presence of lifetime suicidal ideation. Based on these results, we recommend that early screening of psychopathology and suicidal ideation in PLHIV, as well as substituting efavirenz with other ART medications are imperative to promote early psychological intervention, prevent suicide, and improve the overall HIV/AIDS treatment outcome in Indonesia.

## Data Availability

Datasets used in this study are available from the corresponding author (KS) upon request.

## Abbreviations

HIV: Human immunodeficiency virus
AIDS: Acquired immune deficiency syndrome
ART: Antiretroviral therapy
SCL-90: Symptom checklist-90
PLHIV: People living with HIV
CD4: Cluster of differentiation 4
LMICs: Low- and middle-income countries
HICs: High-income countries
