# Correction to: Suicidal ideation, psychopathology and associated factors among HIV-infected adults in Indonesia

**DOI:** 10.1186/s12888-020-02918-0

**Published:** 2020-10-20

**Authors:** Youdiil Ophinni, Adrian, Kristiana Siste, Martina Wiwie, Gina Anindyajati, Enjeline Hanafi, Reza Damayanti, Yoshitake Hayashi

**Affiliations:** 1 Department of Pathology, Graduate School of Medicine, Kobe University, Kobe, Japan; 2 Department of Psychiatry, Faculty of Medicine, Universitas Indonesia, 71 Diponegoro, Central Jakarta, DKI, Jakarta, Indonesia

**Correction to: BMC Psychiatry 20, 255 (2020)**

**https://doi.org/10.1186/s12888-020-02666-1**

Following publication of the original article [1], an error was identified in the Results section of the Abstract.

The updated result is given below and the changes have been highlighted in **bold typeface**.

Results:

**Multivariate analysis identified that a single-point increase in SCL-90 depression symptoms score (AOR 1.26, 95% CI 1.08–1.48,** ***p*** **= 0.004** and efavirenz use (AOR 5.00, 95% CI 1.02–24.6, *p* = 0.048) were significant independent factors related to suicidal ideation.
